# Identification of an RNA binding protein-related gene signature in hepatocellular carcinoma patients

**DOI:** 10.1186/s10020-020-00252-5

**Published:** 2020-12-09

**Authors:** Li Wang, Na Zhou, Jialin Qu, Man Jiang, Xiaochun Zhang

**Affiliations:** Precision Medicine Center of Oncology, The Affiliated Hospital of Qingdao University, Qingdao University, 16 Jiangsu Road, Qingdao, 266003 China

**Keywords:** Gene signature, Nomogram, RNA binding proteins, TCGA database, ICGC database

## Abstract

**Background:**

Hepatocellular carcinoma (HCC) is a common malignant primary cancer with high mortality. Previous studies have demonstrated that RNA binding proteins (RBPs) are involved in the biological processes of cancers, including hepatocellular cancer.

**Methods:**

In this study, we aimed to identify the clinical value of RNA-binding proteins for hepatocellular carcinoma. We obtained gene expression and clinical data of hepatocellular carcinoma patients from the TCGA and ICGC databases. The prognostic value of RBP-related genes in patients with hepatocellular carcinoma and their function were studied by comprehensive bioinformatics analyses. The gene signature of SMG5, EZH2, FBLL1, ZNF239, and IGF2BP3 was generated by univariate and multivariate Cox regression and LASSO regression analyses. We built and verified a prognostic nomogram based on RBP-related genes. The gene signature was validated by the ICGC database. The expression of RBP-related genes was validated by the Oncomine database, the Human Protein Atlas and Kaplan–Meier plotter.

**Result:**

Most RBP-related genes were significantly different in cancer and normal tissues. The survival of patients in the different groups was significantly different. The gene signature showed good performance for predicting the survival of HCC patients by having a better area under the receiver operating characteristic curve than other clinicopathological parameters.

**Conclusion:**

Gene signatures based on RNA-binding proteins can be independent risk factors for hepatocellular carcinoma patients.

## Background

Hepatocellular carcinoma (HCC) is a common cancer with high mortality (Nakano et al. 2020). Cancer of the liver and intrahepatic bile ducts was responsible for an estimated 841,000 new cases and approximately 780,000 deaths in 2018 alone (Park 2015). Moreover, the overall 1- and 3-year survival rates are only 36% and 17%, respectively, in hepatocellular carcinoma patients (El-Serag 2004). Although we have made great progress in radiofrequency ablation, systemic therapy, liver transplantation, targeted therapies, and immunotherapy for treating HCC, the prognosis of HCC remains poor (Zheng 2015). Moreover, patients with the same tumor stage may have different prognoses because of individual differences. Therefore, it is essential to explore alternative biomarkers to predict the prognosis of hepatocellular carcinoma.

RNA binding proteins are a type of protein that can interact with various types of RNAs, including mRNAs, rRNAs, ncRNAs, snRNAs, miRNAs, tRNAs, and snoRNAs (Gerstberger et al. 2014). Currently, approximately 1542 RBP genes have been identified through genome-wide screening of the human genome(Gerstberger et al. 2014). RBPs can regulate post-transcriptional regulation (mRNA stability, RNA processing, splicing, localization, and translation) by binding to their target RNAs to form ribonucleoprotein complexes (Masuda and Kuwano 2019). Post-transcriptional regulation plays a key role in life processes. Therefore, aberrantly deregulated RBPs are closely related to the occurrence and progression of numerous human diseases. Some studies have shown that RBPs are pivotal regulators that regulate the occurrence and progression of cardiovascular diseases by mediating a wide range of post-transcriptional events (Bruin 2017). Previous studies have shown that RBPs are widely expressed in tumor cells, which affects the translation of mRNA into proteins and is involved in carcinogenesis (Pereira et al. 2017; Chatterji and Rustgi 2018). Currently, only a few RBPs have been reported to play key roles in cancer development, such as HuR, AGO2, QKI-5, and ESRP1 (Xie 2019; Zhang 2019; Zong 2014; Jeong 2017). Thus, we will better understand the function of RBPs in cancer through comprehensive analysis.

In this work, we conducted an extensive analysis based on transcript and clinical data obtained from the TCGA and ICGC databases. We applied consensus clustering analysis, least absolute shrinkage and selection operator (LASSO) regression analysis and Cox regression analysis to develop prognostic RBP-related gene signatures. We developed a prognostic model based on RBPs as independent risk factors to predict the prognosis of hepatocellular carcinoma and to suggest therapeutic targets for hepatocellular carcinoma.

## Materials and methods

### Data download and processing

We analyzed the differential expression of RBPs between HCC and adjacent normal tissues using the limma package, with thresholds of false discovery rate (FDR) < 0.05 and a |log2-fold change (FC)|> 2. According to the existing literature, a total of 1542 RBPs were obtained. RNA-Seq transcriptome and clinical data of hepatocellular carcinoma were downloaded from the TCGA database. The expression values at the probe level were converted into the corresponding gene symbol according to the annotation files without further standardization. When several probes matched an identical gene symbol, the mean value was calculated as the expression value of this gene.

### GO and KEGG enrichment analysis

GO enrichment analysis of differential expression of RBPs mainly includes 3 parts: biological processes (BPs), cellular components (CCs), and molecular function (MF) (Ashburner 2000). The KEGG database is an integrated database resource for the biological interpretation of genome sequences and other high-throughput data (Kanehisa 2016). GO and KEGG enrichment analyses were performed using the clusterProfiler package, with thresholds of P and FDR values less than 0.05, indicating statistical significance (Yu 2012).

### Construction of gene signature

Univariate Cox regression analysis was performed on the differential expression of RBPs to obtain RBPs significantly related to survival. Then, we employed least absolute shrinkage and selection operator (LASSO) regression analysis to remove highly correlated survival-related RBPs (Sauerbrei et al. 2007). We identified the prognostic RBPs and their coefficients by multivariate Cox regression analysis, on which we constructed the gene signature. The risk score was calculated as follows: Risk score = $\sum_{i=1}^{n}vi \times ci$ (where *v*_*i*_ is the mean expression of the gene and *c*_*i*_ means the regression coefficient of the gene).

According to the gene signature, a Kaplan–Meier survival curve was plotted to evaluate the high- and low-risk groups by the log-rank test. Moreover, we determined the accuracy of the gene signature by generating receiver operating characteristic (ROC) curves. Independent prognostic analysis was used to predict whether the gene signature could be used as an independent prognostic factor for HCC patients.

### The establishment of RBP the nomogram

Nomograms can predict the likelihood of an event based on the patient’s personal data, such as survival and recurrence. In this study, the establishment of the RBP nomogram was based on the hub RBPs. The predictive accuracy and discriminative value of the nomogram mainly included the concordance index (C-index), AUC and calibration curve (Wang 2013).

### Gene signature validation by the ICGC database

External validation of the gene signature was performed by the International Cancer Genome Consortium (ICGC) database. The risk score of each HCC patient was calculated by the same formula. RNA microarray and clinical data of Japanese HCC patients were downloaded from the ICGC database.

### Verification of the expression and prognostic significance of RBPs

The Oncomine database is a cancer microarray database and web-based data-mining platform that is used to mine cancer gene information (Rhodes 2004). The Oncomine database was applied for differential expression classification of common cancer types and their respective normal tissues as well as clinical and pathological analyses. In addition, The Human Pathology Atlas allowed for the generation of personalized genome-scale metabolic models for cancer patients to identify key genes involved in tumor growth. In this study, we explored the expression of RBPs between HCC tissues and liver tissues using the Oncomine database and The Human Protein Atlas. The prognostic value of the RBPs in HCC was verified by the Kaplan–Meier plotter online tool.

### Statistical analysis

All statistical analyses were performed using the Perl language and R language. LASSO regression analysis and Cox regression analyses were utilized to screen the RBPs related to survival. All significant comparisons were defined as P < 0.05.

## Results

### Differentially expressed RBPs

**Fig. 1:**
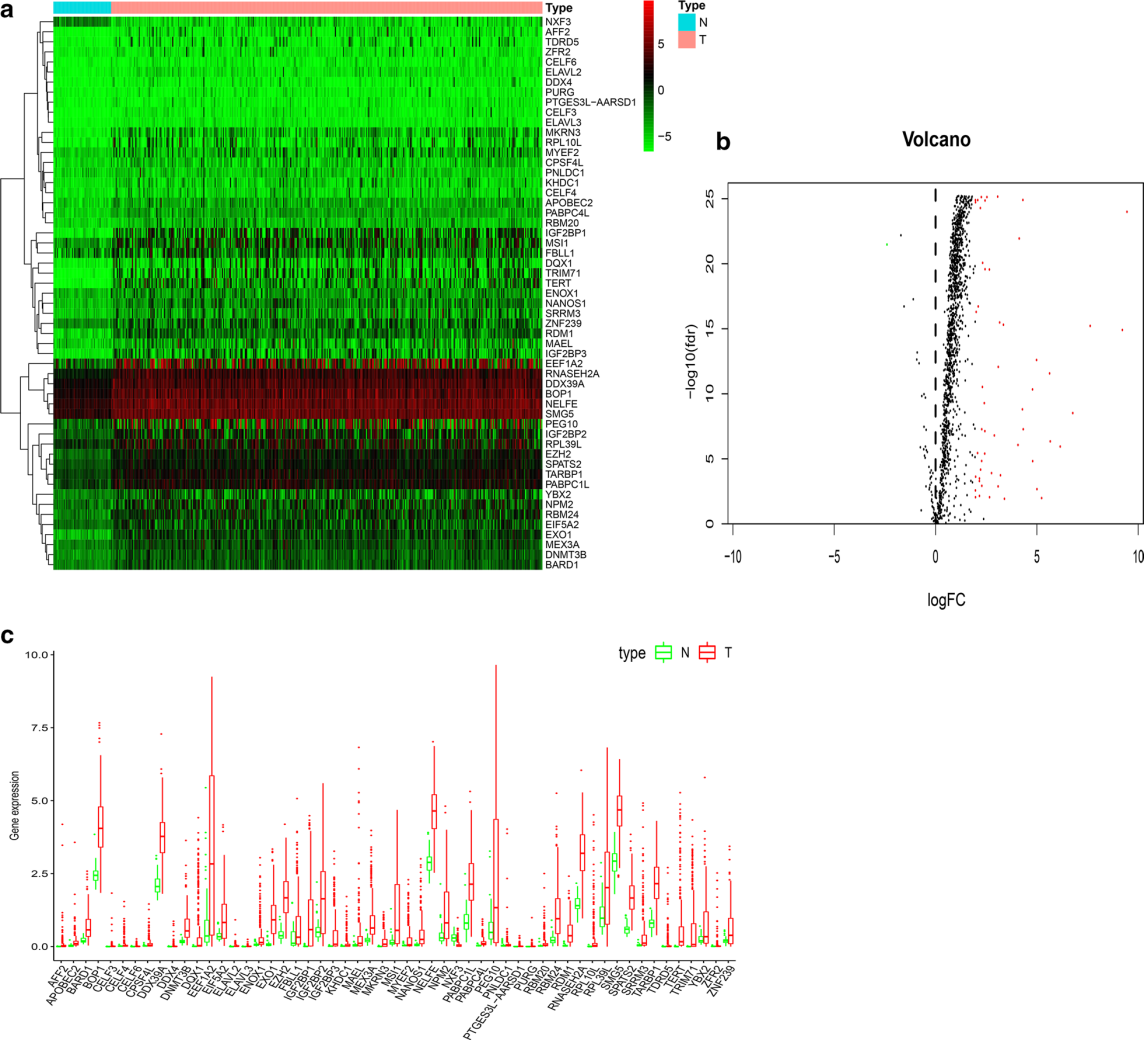
Differentially expressed RBPs between HCC and normal tissues. **a** Heatmap of differentially expressed RBPs. **b** The volcano plot for the differentially expressed RBPs. Red, higher expression; green, lower expression; Black, no difference. **c** The expression patterns of differentially expressed RBPs. Red, tumor tissues; Green, normal tissues

Differentially expressed RBPs were obtained from data analysis (Fig. 1a). All the RBPs were included in the analysis, and 56 RBPs met the screening standard of this study. We visualized the expression pattern of the differentially expressed RBPs using volcano plots and box plots (Fig. 1b, c). The clinical characteristics of the TCGA and ICGC cohorts are shown in Table 1.

**Table 1:** Clinical characteristics of TCGA and ICGC cohorts

Clinical characteristics	TCGA cohorts	ICGC cohorts
Total cases	365	260
Survival status		
Alive	248 (68.0%)	214 (82.3%)
Dead	117 (32.0%)	46 (17.7%)
Age		
< 65	213 (58.4%)	91 (35%)
≥ 65	152 (41.6%)	169 (65%)
Gender		
Male	243 (66.6%)	192 (73.8%)
Female	122 (33.4%)	68 (26.2%)

### GO and KEGG enrichment analyses

**Fig. 2:**
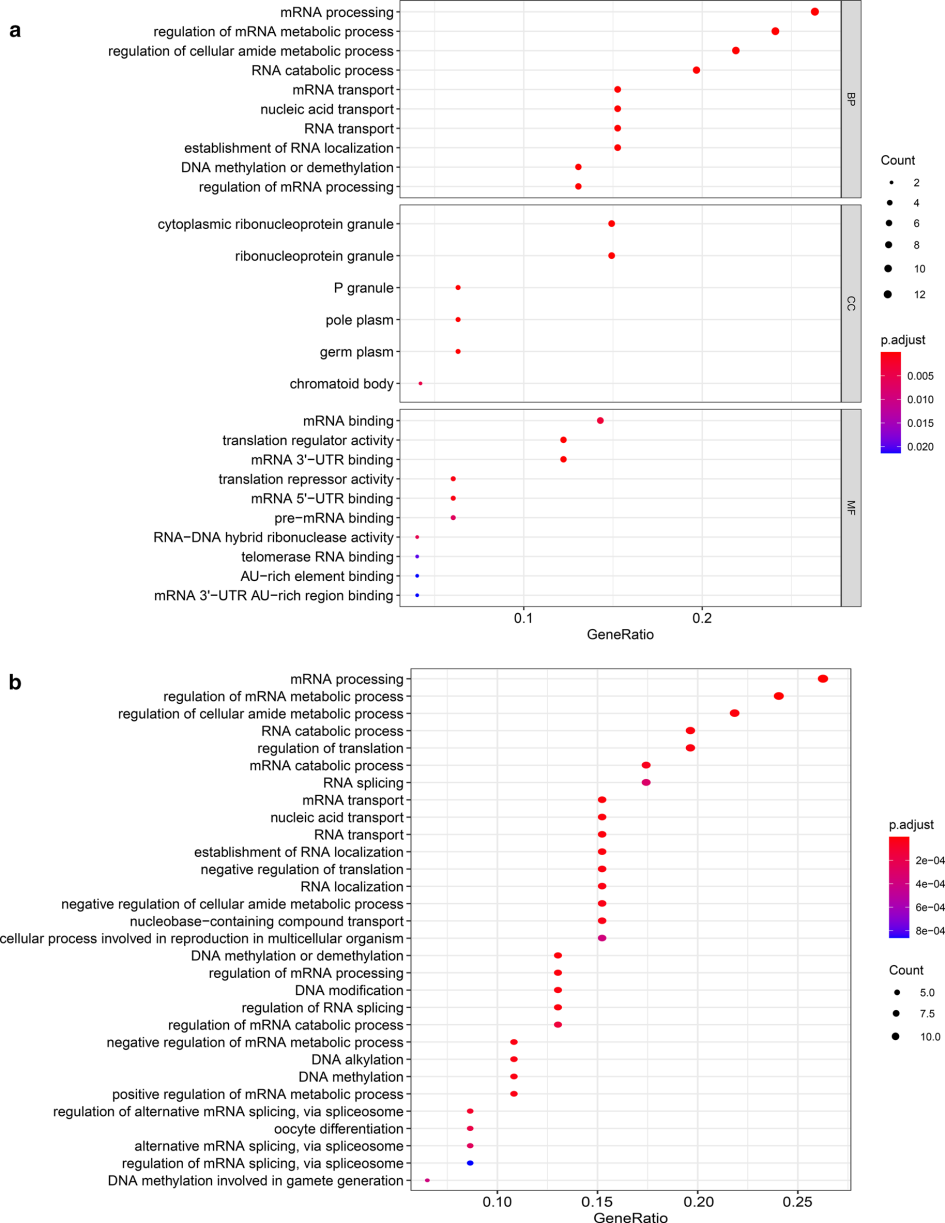
Go and KEGG analysis of differentially expressed RBPs. **a** Bubble plot of enriched GO terms. **b** Bubble plot of KEGG

GO enrichment analysis showed that the differentially expressed RBPs were mainly associated with the BP terms mRNA processing, regulation of mRNA metabolic processes, regulation of cellular amide metabolic processes, and RNA catabolic processes. In addition, the CC terms showed that the RBPs were associated with cytoplasmic ribonucleoprotein granules, ribonucleoprotein granules, P granules, and pole plasms. Moreover, the MF terms mainly included mRNA binding, translation regulator activity, mRNA 3′-UTR binding, and translation repressor activity (Fig. 2a). We also performed KEGG pathway enrichment analysis of mRNA processing, regulation of mRNA metabolic processes, regulation of cellular amide metabolic processes, and RNA catabolic processes (Fig. 2b, c).

### Construction of the gene signature

**Fig. 3:**
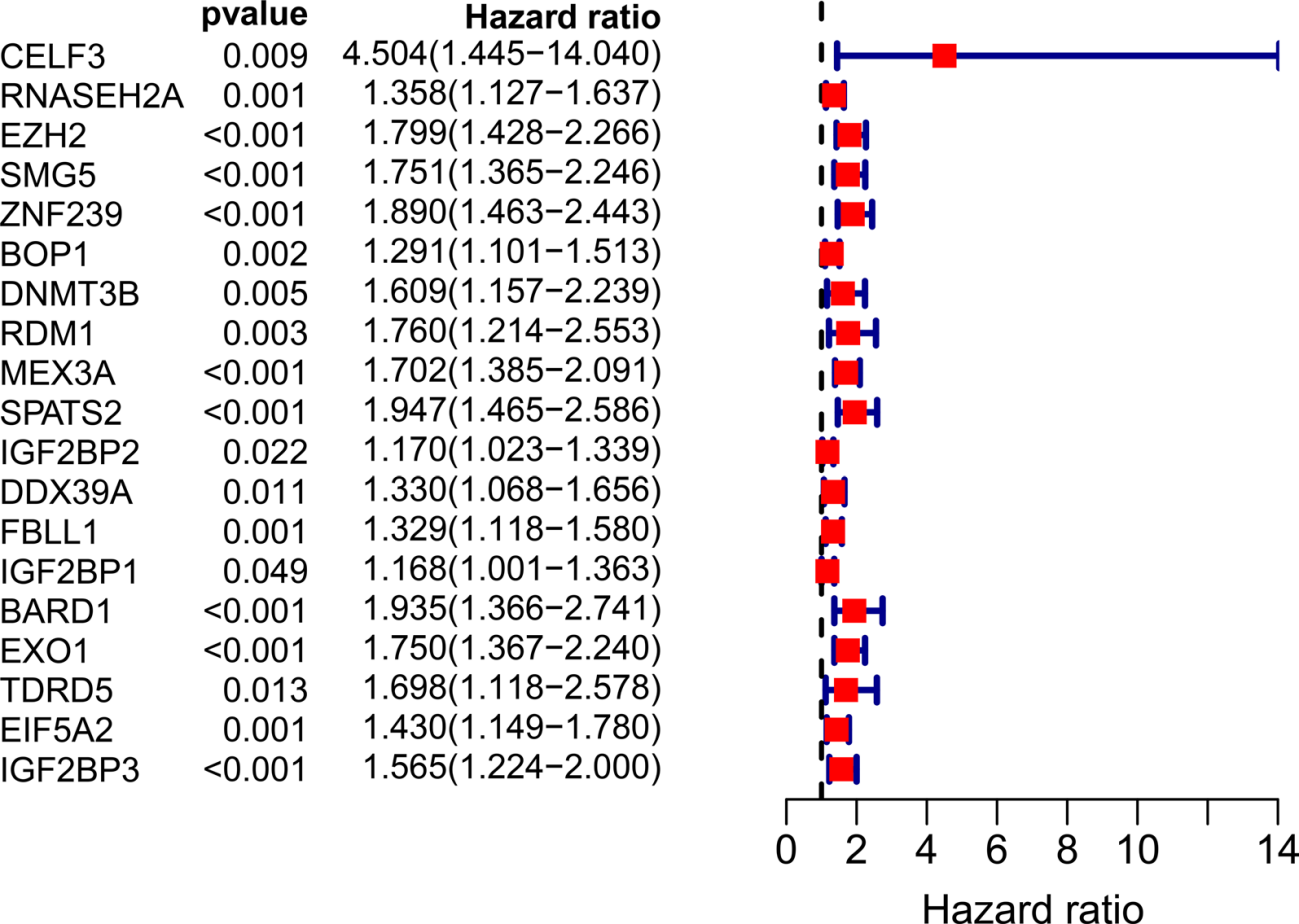
Univariate Cox regression analysis of differentially expressed RBPs

**Fig. 4:**
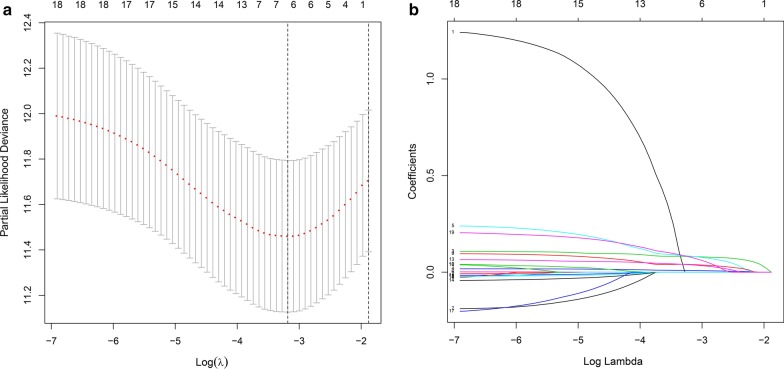
The LASSO regression analysis applied to screening RBPs that optimal used for the construction of the gene signature. **a** Screening of optimal parameter (lambda) at which the vertical lines were drawn. **b** LASSO coefficient profiles of the 6 RBPs with non-zero coefficients determined by the optimal lambda

A total of 19 RBPs were identified as closely related to HCC patient survival by univariate Cox regression analysis (Fig. 3). Then, we removed coexpressed RBP-related genes to prevent data overfitting by LASSO regression analysis (Fig. 4a, b). Finally, 6 RBPs were further submitted to a multivariate Cox proportional hazards model, and 5 candidate RBPs (SMG5, EZH2, FBLL1, ZNF239, and IGF2BP3) were identified to construct the gene signature (Table 2). The risk score of each patient was calculated based on the following formula: risk score = (the mean expression of SMG5*0.013774) + (the mean expression of EZH2*0.095776) + (the mean expression of FBLL1*0.054092) + (the mean expression of ZNF239*0.156661) + (the mean expression of IGF2BP3*0.147735).

**Table 2:** The regression coefficient of five candidate RBPs genes

Gene	Coef	HR	HR.95L	HR.95H	p-value
EZH2	0.095775967	1.100512485	1.020712414	1.186551386	0.012640121
SMG5	0.013773739	1.013869034	1.001508311	1.026382315	0.027751472
ZNF239	0.156660532	1.169598506	1.015670904	1.346854241	0.029559955
FBLL1	0.054092276	1.055582002	1.014381356	1.098456076	0.007747078
IGF2BP3	0.147735119	1.159205805	0.987378077	1.360935724	0.071107802

**Fig. 5:**
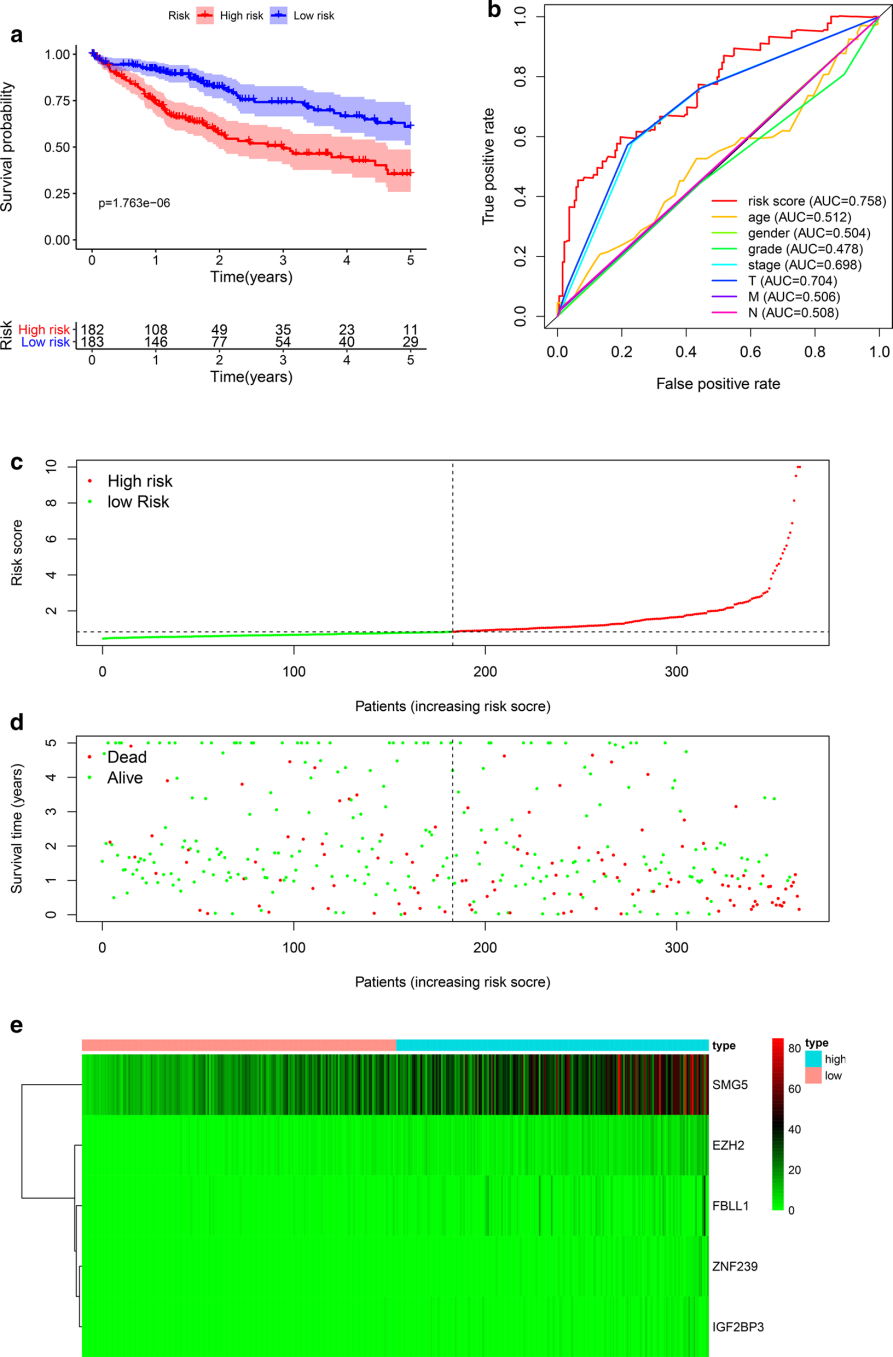
Gene signature of hepatocellular carcinoma patients in TCGA cohort. **a** Kaplan–Meier curve of high-risk and low-risk HCC patients. **b** ROC curve of OS-related gene signature. **c** Risk score distribution of HCC patients with different risks. **d** Scatterplots of HCC patients with different survival status. **e** Heatmap of expression of 5 RBPs in HCC patients

**Fig. 6:**
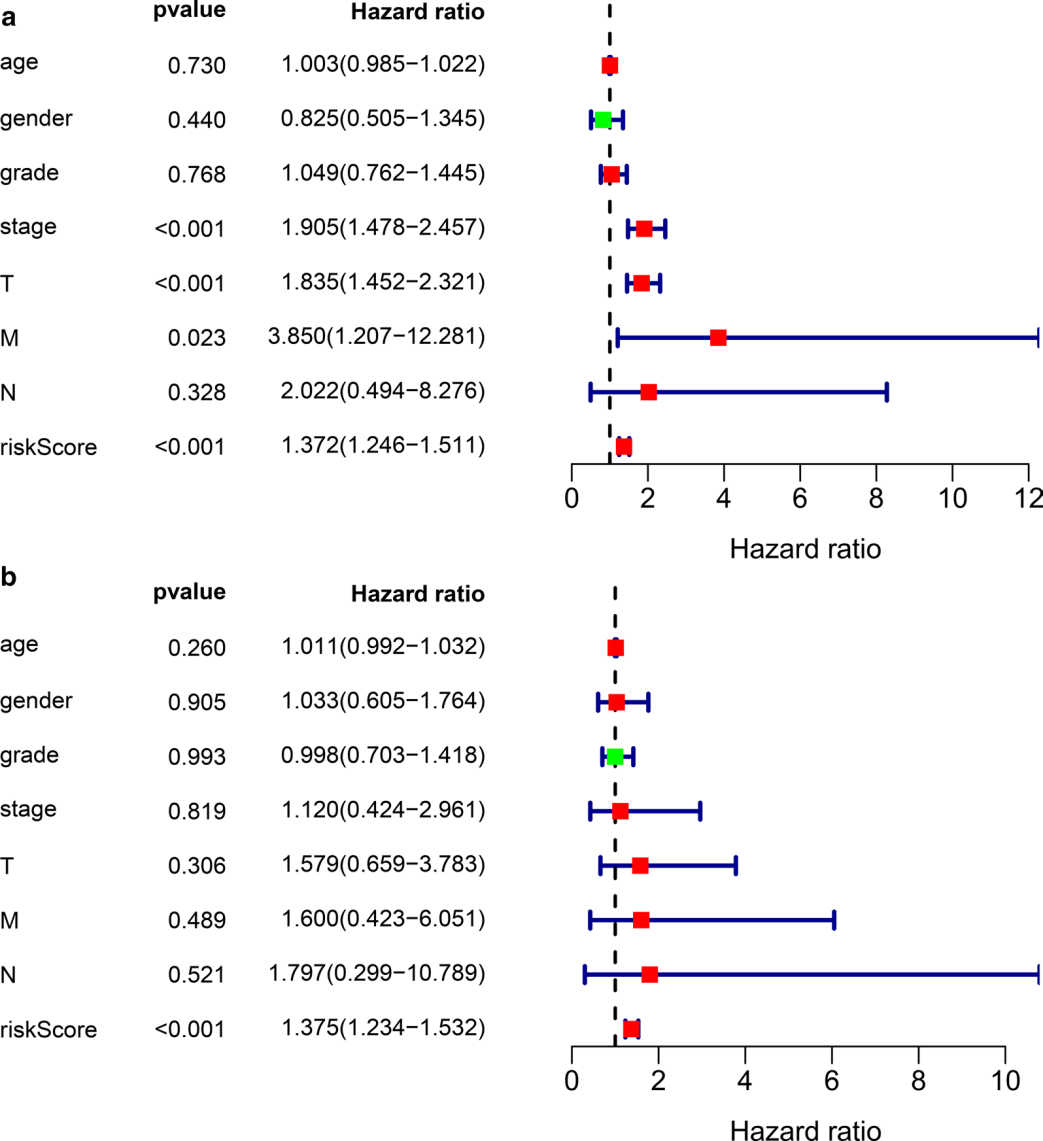
Independent prognostic analysis. **a** Univariate factor independent prognostic analysis. **b** Multivariate factor independent prognostic analysis

All HCC patients were divided into high- and low-risk groups according to the median risk score. The Kaplan–Meier survival curves showed that the low-risk group had a better survival rate than the high-risk group (HR 1.372, 95% CI 1.246–1.511, P < 0.001) (Fig. 5a). In addition, we assessed the accuracy of the 5-OS-related gene signature by constructing a ROC curve, and compared other clinicopathological parameters, the AUC of the risk score was significant (Fig. 5b). Finally, we ranked the HCC patients according to the gene signature to analyze the survival distribution. We identified the mortality rate of HCC patients with their risk scores. Moreover, with the increase in risk score, the mortality rate of patients increased (Fig. 5c, d). We describe the expression level of RBPs with the different risk scores of samples using heat maps (Fig. 5e). The Cox regression analysis showed that the gene signature can be used as an independent prognostic factor for HCC patients (Fig. 6a, b).

### Construction of RBP nomogram

**Fig. 7:**
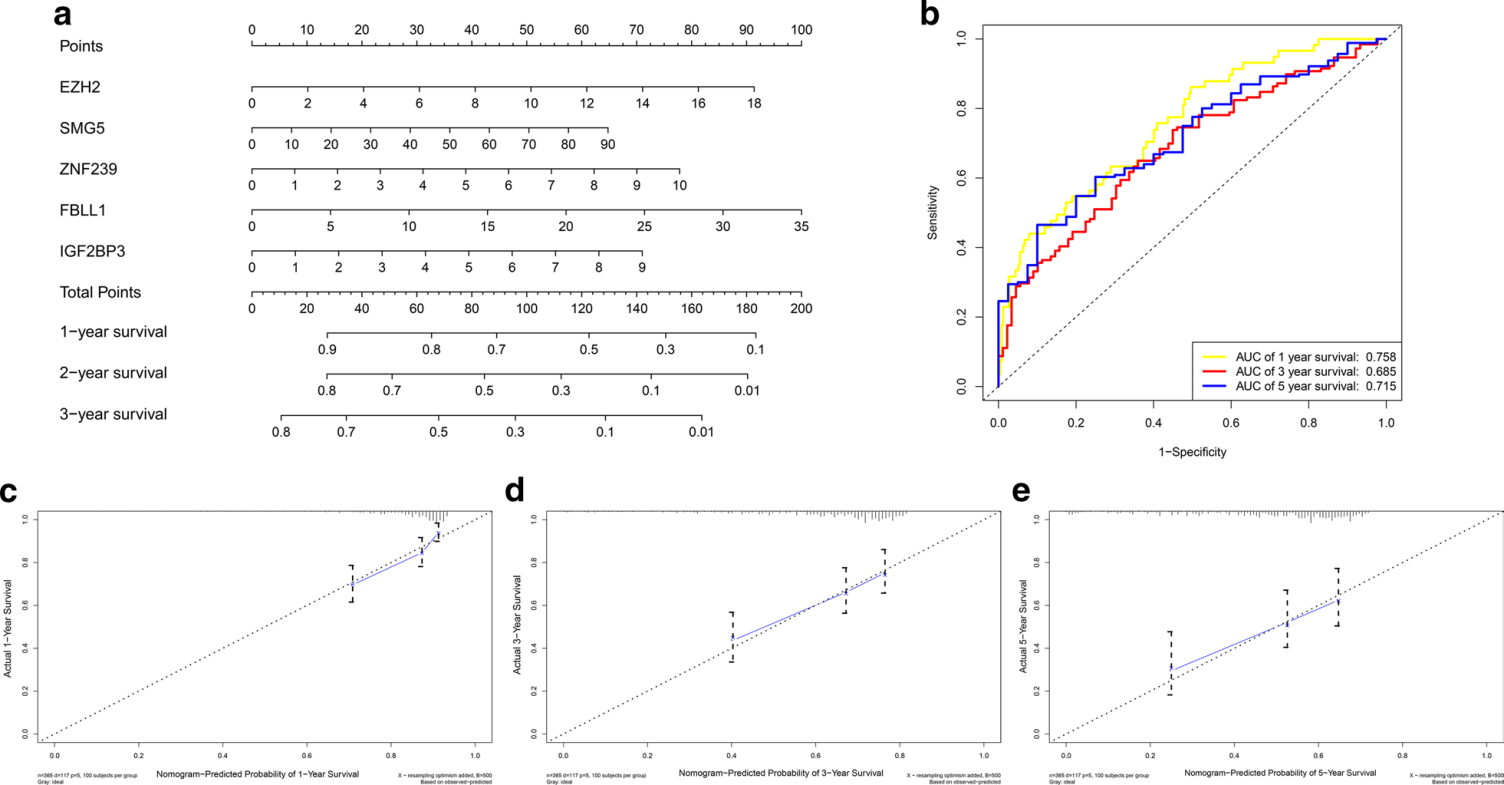
The RBPs nomogram for prediction on survival probability in HCC patients. **a** Development of nomogram for predicting 1-, 3-, and 5-years OS for HCC patients. **b** 1-year,3-year, and 5-year survival ROC of nomogram. **c** The calibration curve for predicting HCC patient 1-year survival. **d** The calibration curve for predicting HCC patient 3-year survival. **e** The calibration curve for predicting HCC patient 5-year survival. X-axis: Nomogram-predicted probability of overall survival; Y-axis: actual overall survival

We established an RBP nomogram to connect the gene signature with 1-year, 2-year, and 3-year survival. We analyzed the RBPs that affect the prognosis of HCC patients and established an RBP nomogram using Cox multivariate analysis. Ultimately, the nomogram included 5 prognostic RBPs (SMG5, EZH2, FBLL1, ZNF239, and IGF2BP3) (Fig. 7a). The C-index of the nomogram for OS prediction was 0.686 (95% CI 0.634–0.738). The 1-year, 3-year and 5-year survival AUCs of the nomogram explained that our nomogram was suitable for clinical application (Fig. 7b). The calibration curve for predicting 1-year, 3-year and 5-year survival also showed that the nomogram was suitable for clinical practice (Fig. 7c–e).

### Validation of the gene signature

**Fig. 8:**
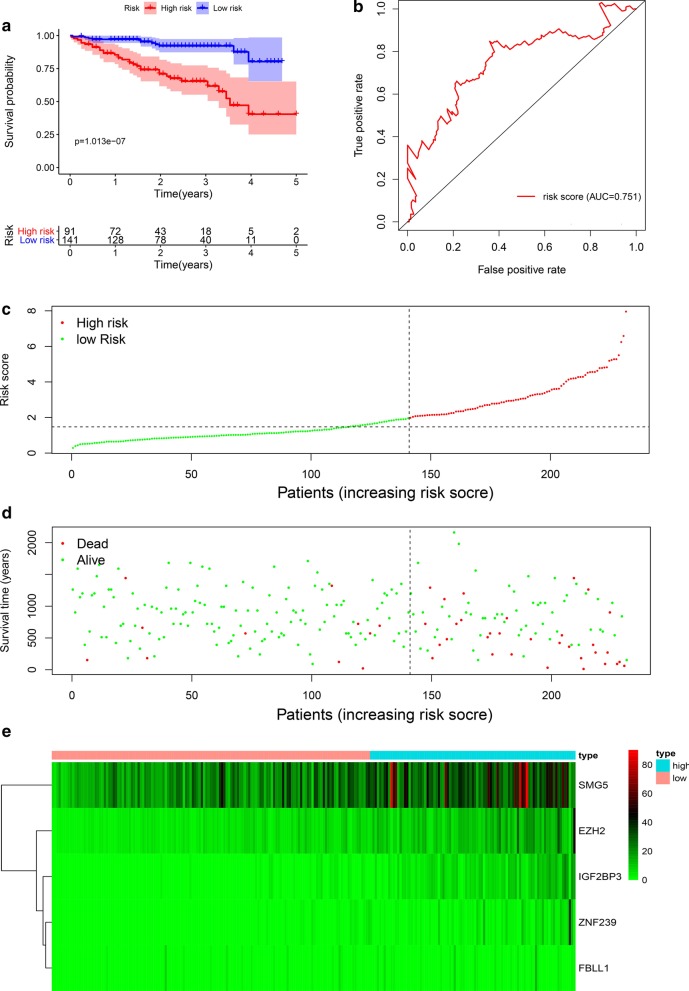
Gene signature of hepatocellular carcinoma patients in ICGC cohort. **a** Kaplan–Meier curve of high-risk and low-risk HCC patients. **b** ROC curve of OS-related gene signature. **c** Risk score distribution of HCC patients with different risks. **d** Scatterplots of HCC patients with different survival status. **e** Heatmap of expression of 5 RBPs in HCC patients

We calculated the risk score of each HCC patient in the ICGC data portal project Liver Cancer-RIKEN, JP (LIRI-JP) as an independent external validation by the same formula. The HCC patients were divided into high- and low-risk groups based on the median risk score. The Kaplan–Meier survival curves show the prognostic value of our gene signature (P < 0.001) (Fig. 8a). In addition, the ROC curve also showed the good ability of the OS-related gene signature to predict the prognosis of HCC patients (Fig. 8b). With the increase in the risk score, the mortality rate of patients increased (Fig. 8c, d). Heat maps were used to describe the expression of RBPs with the different risk scores of samples (Fig. 8e). Therefore, these validation results confirmed the prognostic ability of our gene signature. The sensitivity and specificity of prognostic model was shown in Additional file 1.

### The expression of RBPs in the Oncomine database, human protein Atlas, and Kaplan–Meier plotter

**Fig. 9:**
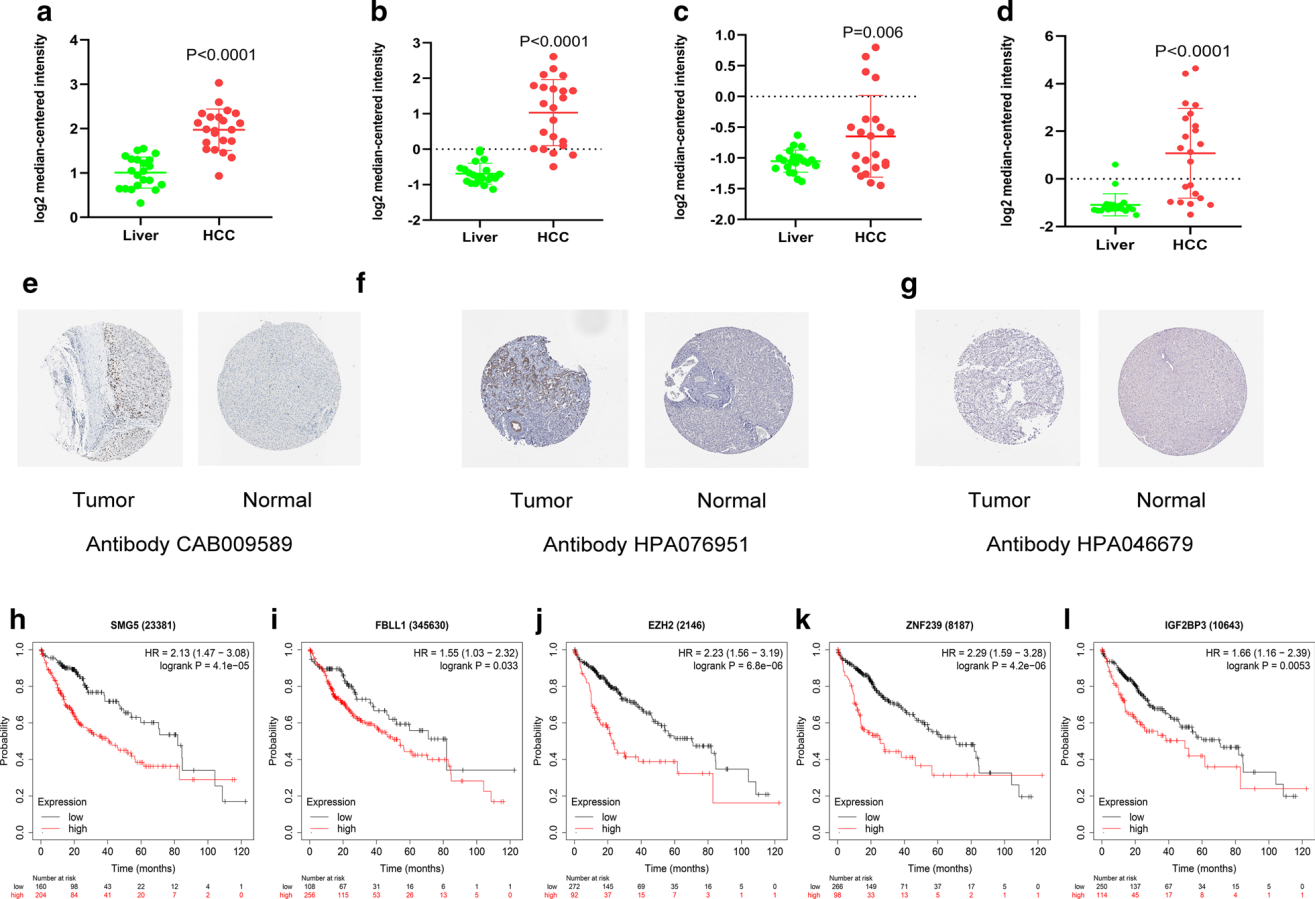
The expression level of SMG5, EZH2, FBLL1, ZNF239, IGF2BP3 in the Oncomine database and The Human Protein Atlas. **a** Expression level of SMG5 in HCC and Liver. **b** Expression level of EZH2 in HCC and Liver. **c** Expression level of ZNF239 in HCC and Liver. **d** Expression level of IGF2BP3 in HCC and Liver. **e** Immunohistochemistry results of EZH2 in HCC (Staining: Medium; Intensity: Moderate; Quantity: 75–25%; Location: Nuclear) and in normal tissue (Staining: Not detected; Intensity: Negative; Quantity: None; Location: None). **f** Immunohistochemistry results of IGF2BP3 in HCC (Staining: high; Intensity: strong; Quantity: 75–25%; Location: Cytoplasmic/membranous) and in normal tissue (Staining: Not detected; Intensity: Negative; Quantity: None; Location: None). **g** Immunohistochemistry results of ZNF239 in HCC (Staining: Not detected; Intensity: Negative; Quantity: None; Location: None) and in normal tissue (Staining: Not detected; Intensity: Negative; Quantity: None; Location: None). **h** Prognostic value of SMG5. **i** Prognostic value of FBLL1. **j** Prognostic value of EZH2. **k** Prognostic value of ZNF293. **l** Prognostic value of IGF2BP3

We analyzed the expression of SMG5, EZH2, FBLL1, ZNF239, and IGF2BP3 in liver cancer using the Oncomine database. The expression levels of SMG5, EZH2, ZNF239, and IGF2BP3 in different hepatocellular carcinomas were higher than those in the normal group in the Roessler Liver (34868_ at), Roessler Liver (203358_s_at), Roessler Liver (206261_at), and Roessler Liver (203819_s_at) studies (Fig. 9a–d). However, FBLL1 was not detected in the Oncomine database.

In addition, we verified the histological levels of SMG5, EZH2, FBLL1, ZNF239, and IGF2BP3 using the Human Protein Atlas database, and the results showed that EZH2 and IGF2BP3 were upregulated in HCC tissues and downregulated in normal tissue (Fig. 9e, f). The histological level of ZNF239 was not significantly different between tumor and normal tissue (Fig. 9g). However, SMG5 and FBLL1 were not detected in the Human Protein Atlas database.

The prognostic significance of SMG5, EZH2, FBLL1, ZNF239, and IGF2BP3 was identified using the Kaplan–Meier plotter server. The results showed that the 5 RBPs were closely related to OS of HCC patients (Fig. 9h–l).

## Conclusion

The carcinogenesis and development of HCC involve a complex regulatory network. Currently, compared to using a single clinicopathological parameter, gathering diverse biomarkers and establishing a gene signature and nomogram are effective ways to predict the prognosis of tumors. RBP dysregulation has been reported in various malignant tumors (Pereira et al. 2017; Wu 2019). The gene signature and nomogram based on RBPs may be more precise than a single clinicopathological parameter.

In this study, we aimed to analyze the relationship between RBPs and the prognosis of HCC patients. First, we downloaded the RNA expression profiles of RBPs from HCC patients from the TCGA database. Then, the results of GO analysis showed that the RBPs were mainly enriched in mRNA processing, regulation of mRNA metabolic processes, RNA catabolic processes, translation regulator activity, and mRNA binding. The KEGG analysis results showed that the ARGs were primarily enriched in mRNA processing, regulation of mRNA metabolic processes, regulation of cellular amide metabolic processes, and RNA catabolic processes. In addition, a total of 56 survival-related RBPs were identified as significantly related to HCC survival by univariate Cox regression analysis. Finally, we determined 5 RBP genes (SMG5, EZH2, FBLL1, ZNF239, and IGF2BP3) and constructed the gene signature by multivariate Cox regression analysis. The gene signature could be an independent prognostic biomarker for HCC patients.

Nomograms, a user-friendly graphical composite model, have been shown to be more accurate than conventional staging systems for predicting prognosis in various cancers (Sternberg 2006). A nomogram can predict the likelihood of an event based on the patient’s personal data, such as survival and recurrence. To make our gene signature achieve a more credible and valuable prediction power for clinical application, a nomogram including SMG5, EZH2, FBLL1, ZNF239, and IGF2BP3 was developed to assess the individualized survival risk of patients and demonstrated satisfactory discrimination.

SMG5 nonsense-mediated mRNA decay factor (SMG5) is involved in nonsense-mediated mRNA decay (Ohnishi 2003). Previous studies have indicated that SMG5 is an important nonsense-mediated mRNA decay factor (Jin 2016). Enhancer of zeste 2 polycomb repressive complex 2 subunit (EZH2) is involved in various biological processes (Comet 2016; Crea 2011). Increasing research has indicated that EZH2 is widely associated with carcinomas, such as hepatocellular carcinoma, colorectal cancer, melanoma and neuroendocrine tumors (Xiao 2019a; Di 2019; Emran 2019; Faviana 2019). Recently, a study showed that the epigenetic modifier EZH2 can suppress the expression of the immune checkpoint inhibitor PD-L1 by directly upregulating the level of the promoter H3K27me3 for CD274 and IRF1 in hepatoma cells and may serve as a potential therapeutic target for immunotherapy for treating immune-activated HCC (Xiao 2019b). Insulin-like growth factor 2 mRNA binding protein 3 (IGF2BP3), a member of the IMP family, plays an important role in cell migration in early embryogenesis (Gong et al. 2014; Vikesaa 2006). IGF2BP3 has gained considerable interest as a cancer-associated protein. A previous study reported IGF2BP3 overexpression in a variety of types of human cancers (Burdelski 2018). These studies suggest that IGF2BP3 may represent a valuable prognostic marker in human cancer.

The gene signature was validated by the ICGC cohort. The expression of SMG5, EZH2, FBLL1, ZNF239, and IGF2BP3 was determined using the Oncomine database, the Human Protein Atlas and Kaplan–Meier plotter. Post-transcriptional regulation is a dynamic and continuous process. It is still not clear if analyzing changes in RNA binding protein‑related genes is sufficient to reflect RBPs. Therefore, there are some limitations in our work. First, there are no experimental studies regarding the link between expression data and functional autophagy states to verify our results. Second, there are not enough clinical studies to confirm our results.

In conclusion, our study constructed a gene signature based on RNA binding protein‑related genes for HCC patients. Moreover, we further established a prognostic nomogram for hepatocellular carcinoma patients. Our gene signature and nomogram have great value for application in clinical practice. However, these RBPs still need further research to explore whether they may be helpful for molecular-targeted therapy of liver hepatocellular carcinoma.

## Supplementary Information

**Additional File 1.** The sensitivity and specificity of prognostic model.

## Data Availability

Not applicable.

## Abbreviations

HCC: Hepatocellular carcinoma
RBPs: RNA binding proteins
TCGA: The genomic data commons data portal and the cancer genome atlas database
ICGC: International Cancer Genome Consortium
GO: Gene ontology database
KEGG: The Kyoto Encyclopedia of Genes and Genomes database
ROC curve: Receiver operating characteristic curve
AUC: Area under curve

## References

[CR1] Ashburner M (2000). Gene ontology: tool for the unification of biology. The Gene Ontology Consortium. Nat Genet.

[CR2] Burdelski C (2018). IMP3 overexpression occurs in various important cancer types and is linked to aggressive tumor features: a tissue microarray study on 8,877 human cancers and normal tissues. Oncol Rep.

[CR3] Chatterji P, Rustgi AK (2018). RNA binding proteins in intestinal epithelial biology and colorectal cancer. Trends Mol Med.

[CR4] Comet I (2016). Maintaining cell identity: PRC2-mediated regulation of transcription and cancer. Nat Rev Cancer.

[CR5] Crea F (2011). Pharmacologic disruption of Polycomb Repressive Complex 2 inhibits tumorigenicity and tumor progression in prostate cancer. Mol Cancer.

[CR6] de Bruin RG (2017). Emerging roles for RNA-binding proteins as effectors and regulators of cardiovascular disease. Eur Heart J.

[CR7] Di W (2019). Long noncoding RNA SNHG14 facilitates colorectal cancer metastasis through targeting EZH2-regulated EPHA7. Cell Death Dis.

[CR8] El-Serag HB (2004). Hepatocellular carcinoma: recent trends in the United States. Gastroenterology.

[CR9] Emran AA (2019). Targeting DNA Methylation and EZH2 activity to overcome melanoma resistance to immunotherapy. Trends Immunol.

[CR10] Faviana P (2019). EZH2 expression in intestinal neuroendocrine tumors. Appl Immunohistochem Mol Morphol.

[CR11] Gerstberger S, Hafner M, Tuschl T (2014). A census of human RNA-binding proteins. Nat Rev Genet.

[CR12] Gong Y, Woda BA, Jiang Z (2014). Oncofetal protein IMP3, a new cancer biomarker. Adv Anat Pathol.

[CR13] Jeong HM (2017). ESRP1 is overexpressed in ovarian cancer and promotes switching from mesenchymal to epithelial phenotype in ovarian cancer cells. Oncogenesis.

[CR14] Jin Y (2016). MicroRNA 433 regulates nonsense-mediated mRNA decay by targeting SMG5 mRNA. BMC Mol Biol.

[CR15] Kanehisa M (2016). KEGG as a reference resource for gene and protein annotation. Nucleic Acids Res.

[CR16] Masuda K, Kuwano Y (2019). Diverse roles of RNA-binding proteins in cancer traits and their implications in gastrointestinal cancers. Wiley Interdiscip Rev RNA.

[CR17] Nakano S (2020). Recent advances in immunotherapy for hepatocellular carcinoma. Cancers.

[CR18] Ohnishi T (2003). Phosphorylation of hUPF1 induces formation of mRNA surveillance complexes containing hSMG-5 and hSMG-7. Mol Cell.

[CR19] Park JW (2015). Global patterns of hepatocellular carcinoma management from diagnosis to death: the BRIDGE Study. Liver Int.

[CR20] Pereira B, Billaud M, Almeida R (2017). RNA-binding proteins in cancer: old players and new actors. Trends Cancer.

[CR21] Rhodes DR (2004). ONCOMINE: a cancer microarray database and integrated data-mining platform. Neoplasia.

[CR22] Sauerbrei W, Royston P, Binder H (2007). Selection of important variables and determination of functional form for continuous predictors in multivariable model building. Stat Med.

[CR23] Sternberg CN (2006). Are nomograms better than currently available stage groupings for bladder cancer?. J Clin Oncol.

[CR24] Vikesaa J (2006). RNA-binding IMPs promote cell adhesion and invadopodia formation. EMBO J.

[CR25] Wang Y (2013). Prognostic nomogram for intrahepatic cholangiocarcinoma after partial hepatectomy. J Clin Oncol.

[CR26] Wu Y (2019). HPV shapes tumor transcriptome by globally modifying the pool of RNA binding protein-binding motif. Aging (Albany NY).

[CR27] Xiao G (2019). EZH2 negatively regulates PD-L1 expression in hepatocellular carcinoma. J Immunother Cancer.

[CR28] Xiao G (2019). EZH2 negatively regulates PD-L1 expression in hepatocellular carcinoma. J Immunother Cancer.

[CR29] Xie M (2019). The long intergenic non-protein coding RNA 707 promotes proliferation and metastasis of gastric cancer by interacting with mRNA stabilizing protein HuR. Cancer Lett.

[CR30] Yu G (2012). clusterProfiler: an R package for comparing biological themes among gene clusters. OMICS.

[CR31] Zhang H (2019). Acetylation of AGO2 promotes cancer progression by increasing oncogenic miR-19b biogenesis. Oncogene.

[CR32] Zheng Z (2015). Adjuvant chemotherapy for patients with primary hepatocellular carcinoma: a meta-analysis. Int J Cancer.

[CR33] Zong FY (2014). The RNA-binding protein QKI suppresses cancer-associated aberrant splicing. PLoS Genet.

