# Supplementary material for: Identification of an RNA binding protein-related gene signature in hepatocellular carcinoma patients
Source: Mol Med. 2020 Dec 9;26:125. doi: 10.1186/s10020-020-00252-5 (PMC7727152; doi:10.1186/s10020-020-00252-5)
Supplement: Supplementary file 1 — Additional File 1. The sensitivity and specificity of prognostic model. [file 10020_2020_252_MOESM1_ESM.docx]

Supplementary file 1. The sensitivity and specificity of prognostic model

|  | Condition positive | Condition negative |
| --- | --- | --- |
| Prediction positive | 76 | 106 |
| Prediction negative | 41 | 142 |
